# Reviewing independent access to HIV testing, counselling and treatment for adolescents in HIV-specific laws in sub-Saharan Africa: implications for the HIV response

**DOI:** 10.7448/IAS.20.1.21456

**Published:** 2017-08-11

**Authors:** Patrick M. Eba, HyeYoung Lim

**Affiliations:** ^a^ Community Support, Social Justice and Inclusion Department, Joint United Nations Programme on HIV/AIDS, Geneva, Switzerland; ^b^ School of Law, University of KwaZulu-Natal, Pietermaritzburg, South Africa; ^c^ The Global Fund to Fight AIDS, Tuberculosis and Malaria, Geneva, Switzerland

**Keywords:** Adolescents, consent, age, legislation, HIV-specific law, independent, evolving capacity

## Abstract

**Introduction**: AIDS is a leading cause of death among adolescents in sub-Saharan Africa. Yet, legal, policy and social barriers continue to restrict their access to HIV services. In recent years, access to independent HIV testing and treatment for adolescents has gained increased attention. The 2013 WHO Guidance on HIV testing and counselling and care for adolescents living with HIV (WHO Guidance) calls for reviewing legal and regulatory frameworks to facilitate adolescents’ access to comprehensive HIV services. As of 31 March 2017, some 28 countries in sub-Saharan Africa have adopted HIV-specific legislation. But there is limited understanding of the provisions of these laws on access to HIV services for adolescents and their implication on efforts to scale up HIV prevention, testing, treatment and care among this population.

**Methods**: A desk review of 28 HIV-specific laws in sub-Saharan Africa complemented with the review of HIV testing policies in four countries using human rights norms and key public health recommendations from the 2013 WHO Guidance. These recommendations call on countries to (i) lower the age of consent to HIV testing and counselling and allow mature adolescents who have not reached the age of consent to independently access HIV testing, (ii) ensure access to HIV counselling for adolescents, (iii) protect the confidentiality of adolescents living with HIV and (iv) facilitate access to HIV treatment for adolescents living with HIV.

**Results**: Most HIV-specific laws fail to take into account human rights principles and public health recommendations for facilitating adolescents’ access to HIV services. None of the countries with HIV-specific laws has adopted all four recommendations for access to HIV services for adolescents. Discrepancies exist between HIV laws and national policy documents. Inadequate and conflicting provisions in HIV laws are likely to hinder access to HIV testing, counselling and treatment for adolescents.

**Conclusions**: Efforts to end legal barriers to access to HIV services for adolescents in sub-Saharan Africa should address HIV-specific laws. Restrictive provisions in these laws should be reformed, and their protective norms effectively implemented including by translating them into national policies and ensuring sensitization and training of healthcare workers and communities. This study reiterates the need for action in all countries across Africa and beyond to review their laws and policies to create an enabling environment to accelerate access to HIV prevention, testing and treatment services for adolescents.

## Introduction

Adolescence (10–19 years) is a period of dynamic transitions to adulthood characterized by rapid cognitive and physical changes, including sexual and reproductive maturation [1]. It is a time of important transformations in adolescents’ sense of identity and their relationship with their body, peers and social environment that influence their sexuality, desires and other explorative behaviours [2]. Adolescence is usually the period where many people have sex for the first time. In the absence of knowledge, skills and health services in this period of transition, adolescents may be at enhanced risk of HIV and other sexually transmitted infections [3].

Although AIDS-related deaths fell by 30% globally between 2005 and 2012, they have increased by 50% among adolescents [4]. More than 80% of the 2 million adolescents living with HIV worldwide are in sub-Saharan Africa [4]. Access to HIV services among this population remains worryingly low [5]. Globally, only 10% of young men and 15% of young women are aware of their HIV status [4]. Coverage of antiretroviral treatment among adolescents is largely inadequate, resulting in AIDS being a leading cause of death among this population in sub-Saharan Africa [5].

Access to HIV services is particularly limited for adolescents who belong to key populations at higher risk of HIV infection, including adolescents who engage in same-sex sexual relations or use drugs [4,5]. For these young key populations, general vulnerabilities and barriers affecting adolescents are further compounded by stigma, discrimination and other human rights violations linked to punitive laws and practices punishing their sexual practices, behaviour or circumstances [6].

Evidence and data showing that the AIDS response is failing adolescents have recently prompted calls for facilitating their independent (autonomous) and non-discriminatory access to evidence-informed HIV prevention, testing and treatment services. In its final report, the Global Commission on HIV and the Law recommended that states address legal and policy barriers to access to HIV testing and treatment services for children [7]. Similarly, the 2013 WHO Guidance on HIV testing and counselling and care for adolescents living with HIV (WHO Guidance) urged countries to review legal and regulatory frameworks to facilitate adolescents’ access to comprehensive HIV services [4]. Further, the UNAIDS and Lancet Commission called among others for addressing legal and policy barriers to adolescent and young people’s access to HIV services as a necessary measure to advance efforts to end the AIDS epidemic as a public health threat by 2030 [8].

Global and regional human rights norms and principles require states to ensure that adolescents receive voluntary, non-discriminatory and confidential services with due attention to their best interests and evolving capacity (Table 1). In particular, states must set “a minimum age for sexual consent … and the possibility of medical treatment without parental consent” [1]. They must also ensure that the principles of the five “Cs” (Consent, Confidentiality, Counselling, Correct test results and Connections to treatment, care and prevention services) which underpin HIV testing and counselling services in general are upheld for adolescents [4,9].

**Table 1.** HIV services and adolescents: the human rights framework

Key human rights principles and norms	Applicable treaties and other binding instruments	General comments addressing the norm	Concluding observations (examples)
Health	UDHR Art. 25; CRC Art. 24; ICESCR Art. 12; CEDAW Art. 12; ACHPR Art. 16; ACRWC Art. 14; Maputo Protocol Art. 14	CRC GC No. 15 (2013); CRC GC No. 4 (2003); CRC GC No. 3 (2003); CESCR GC No. 14 (2000)	“… Improve access to high-quality, age-appropriate HIV/AIDS, sexual and reproductive health services, including by providing for a minor to undergo HIV treatment on a voluntary basis without the consent of a legal administrator or guardian …” CRC, Mauritius (2015)
Non-discrimination	UDHR Art. 1 and 2; CRC Art. 2; ICCPR Art. 2(1) and Art. 3; ICESCR Art. 2(2) and Art. 3; CEDAW Art. 1 and 2; ACHPR Art. 2 and 18; ACHPR Art. 3; Maputo Protocol Art. 2	CRC GC No. 4 (2003) para 2; CESCR GC No. 20 (2009); CCPR GC No. 18 (1989)	“… to eliminate stereotypes and practices that discriminate against girls …” CRC, Eritrea (2015) “… enact a general law against discrimination with a view to incorporating the prohibition of discrimination included in the Covenant…” CCPR, Côte d’Ivoire (2015)
Best interests of the child	CRC Art 3(1); ACRWC Art. 20	CRC GC No. 14 (2013)	“… develop procedures and criteria to provide guidance to all relevant persons in authority for determining the best interests of the child in every area and for giving those interests due weight as a primary consideration …” CRC, Congo (2014)
Evolving capacity of the child and right to be heard and freedom of expression	UDHR Art. 19; ICCPR 19(2); CRC Art. 12(1), (2) & 13; ACHPR Art. 9(2); ACRWC Art. 7	CRC GC No. 4 (2003); CRC GC No. 12, para 80, 81; CCPR GC No. 10 (1983) & No. 34 (2011)	“… promote and facilitate … respect for views of children and their participation in all matters affecting them in accordance with their evolving capacity”. CRC, Côte d’Ivoire (2001)
Education and information	ICESCR Art. 13; CRC Art. 17, 28 & 29; CEDAW 10; ACHPR Art. 9(2) & 17; Maputo Protocol Art. 12	CRC GC No. 3 (2003) para 22; CRC GC No. 12, para 82; CESCR GC No. 22 (2016)	“… include initiatives to provide education and services to adolescents on reproductive health with information on preventing HIV/AIDS and STIs”. CRC, Guinea Bissau (2013)
Prohibition of harmful cultural practices	CRC Art. 24(3); CEDAW Art. 5(a); ACRWC Art. 21; Maputo Protocol Art. 5;	Joint CRC & CEDAW GC Harmful Practices	“… modify or eliminate negative cultural practices and stereotypes that are harmful to and discriminatory against, women”. CRC, Niger (2009)
Prohibition of torture, inhumane and degrading treatment	UDHR Art. 5; ICCPR Art. 7; CRC Art. 37(a); ACRWC Art. 16	CRC GC No. 8 (2006); CCPR GC No. 20 (2000)	“… review its legislation in order to ensure that infliction of torture or cruel, inhuman or degrading treatment or punishment upon children is considered as an aggravating factor”. CRC, Tunisia (2010)
Privacy (including confidentiality)	UDHR Art. 12; CRC Art. 16(1); ACRWC Art. 10	CRC GC No. 4, para 7; CRC GC No. 3, para 24; CCPR GC 17 (1988)	“… provide for system of voluntary testing for HIV/AIDS with full respect for right to privacy and confidentiality”. CRC, Benin (2006)

UDHR: Universal Declaration on Human Rights; ICCPR: International Covenant on Civil and Political Rights; ICESCR: the International Covenant on Economic Social and Cultural Rights; CRC: Convention on the Rights of the Child; CEDAW, Convention on the Elimination of All forms of Discrimination Against Women; ACHPR: African Charter on Human and Peoples’ Rights; Maputo Protocol: Protocol to the African Charter on Human and Peoples’ Rights on the Rights of Women in Africa; ACRWC: African Charter on the Rights and Welfare of the Child; GC: general comment.

As of 31 March 2017, 28 countries in sub-Saharan Africa have adopted HIV-specific laws with the aim to create an enabling environment for the AIDS response, including for adolescents (see Table 2) [10]. Unlike policy documents, which are not binding, laws obligate national actors, including public health institutions, to abide by their stipulations and to take specific measures to ensure implementation. This article reviews the normative content of HIV-specific laws in sub-Saharan Africa on access to HIV testing, counselling and treatment for adolescents. It examines these laws against four key recommendations in the WHO Guidance, namely (i) lower the age of consent to HIV testing and counselling and allow mature adolescents who have not reached the age of consent to independently access HIV testing, (ii) ensure access to HIV counselling for adolescents, (iii) protect the confidentiality of adolescents living with HIV and (iv) facilitate access to HIV treatment for adolescents living with HIV (Box 1).

**Table 2.** HIV-specific laws in sub-Saharan Africa (as of 31 March 2017)

Country	Title of HIV-specific law
1. Angola	● Lei No 8/04 sobre o Virus da Immunodeficiência Humana (VIH) e a Sindroma de Immunodeficiência Adquirida (SIDA), 2004
2. Benin	● Loi No 2005-31 du 5 Avril 2006 portant prévention, prise en charge et contrôle du VIH/SIDA, 2006
3. Burkina Faso	● Loi No 030-2008/AN portant lutte contre le VIH/SIDA et protection des droits des personnes vivant avec le VIH/SIDA, 2008
4. Burundi	● Loi No 1/018 du 12 Mai 2005 portant protection juridique des personnes infectées par le Virus de l’Immunodéficience Humaine et des personnes atteintes du Syndrome Immunodéficience Acquise, 2005
5. Cape Verde	● Lei No 19/VII/2007, 2007
6. Central African Republic	● Loi 06.030 de 2006 fixant les droits et obligations des personnes vivant avec le VIH/SIDA, 2006
7. Chad	● Loi No 19/PR/2007 du 15 Novembre 2007 portant lutte contre VIH/SIDA/IST et protection des droits des personnes vivant avec le VIH/SIDA, 2007
8. Comoros	● Loi N° 14-011/AU du 21 avril 2014, relative aux droits des personnes vivant avec le VIH et leur implication dans la réponse nationale, 2014
9. Congo	● Loi No 30 - 2011 du 3 juin 2011 portant lutte contre le VIH et le SIDA et protection des droits des personnes vivant avec le VIH, 2011
10. Côte d’Ivoire	● Loi n° 2014-430 du 14 juillet 2014 portant régime de prévention, de protection et de répression en matière de lutte contre le VIH et le SIDA, 2014
11. Democratic Republic of Congo	● Loi No 08/011 du 14 Juillet 2008 portant protection des droits des personnes vivant avec le VIH/SIDA et des personnes affectées, 2008
12. Equatorial Guinea	● Ley No 3/2005 sobre la prevención y la lucha contra las infecciones de transmisión sexual (ITS), el VIH/SIDA y la defensa de los derechos de las personas afectadas, 2005
13. Gambia	● HIV and AIDS Prevention and Control Act, No 4 of 2015
14. Guinea	● Ordonnance No 056/2009/PRG/SGG portant amendement de la loi L/2005/025/AN du 22 Novembre 2005 relative à la prévention, la prise en charge et le contrôle du VIH/SIDA en République de Guinée, 2009
15. Guinea Bissau	● Loi n° 5/2007 du 10 septembre 2007 de la prévention, du traitement et du contrôle du VIH/sida, 2007
16. Kenya	● HIV and AIDS Prevention and Control Act, No 14 of 2006
17. Liberia	● An Act to Amend the Public Health Law, Title 33, Liberian Code of Laws Revised (1976) to Create New Chapter 18 Providing for the Control of Human Immunodeficiency Virus (HIV) and Acquired Immunodeficiency Syndrome (AIDS), 2010
18. Madagascar	● Loi No 2005-040 du 20 Février 2006 sur la lutte contre le VIH/SIDA et la protection des droits des personnes vivant avec le VIH/SIDA), 2006
19. Mali	● Loi No 6-028 du 29 Juin 2006 fixant les règles relatives à la prévention, à la prise en charge et au contrôle du VIH/SIDA, 2006
20. Mauritania	● Loi No 2007-042 relative à la prévention, la prise en charge et le contrôle du VIH/SIDA, 2007
21. Mauritius	● HIV and AIDS Act, No 31 of 2006
22. Mozambique	● Lei No 19/2014 Lei de Protecçao da Pessao, do trabalhador e do Candidato e Emprego Vivendo com VIH e SIDA, 2014
23. Niger	● Loi No 2015-30 du 26 Mai 2015 relative à la prévention, la prise en charge et le contrôle du Virus de d’Immunodéficience Humaine (HIV)
24. Senegal	● Loi n° 2010-03 du 9 avril 2010 relative au VIH/SIDA, 2010
25. Sierra Leone	● The National HIV and AIDS Commission Act of 2011
26. Tanzania	● HIV and AIDS (Prevention and Control) Act, No 28 of 2008
27. Togo	● Loi No 2010-018 du 31 Décembre 2010 modifiant la loi No 2005 - 012 du 14 Décembre 2005 portant protection des personnes en matière de VIH/SIDA, 2010
28. Uganda	● HIV Prevention and Control Act of 2014

In four countries (Burkina Faso, Chad, Kenya and Tanzania), the article further provides a comparative analysis of the provisions of HIV-specific laws against those of existing policies and guidelines relating to HIV testing for adolescents. In these four countries, the study aims to ascertain whether the provisions in HIV-specific laws are translated into policy guidance for HIV implementers and health workers. In doing so, this analysis goes beyond the mere existence of legal provisions on HIV testing, counselling and treatment for adolescents. It assesses whether these provisions are in line with the best available human rights and public health recommendations.

The overall aim of this study is to contribute to better knowledge and understanding of the provisions of HIV-specific laws relating to access to HIV services for adolescents, as well as the implication of these provisions on efforts to scale up HIV testing, treatment and care for adolescents. It is expected that identifying progress and barriers in HIV-specific laws will inform law and policy reform and implementation to facilitate access to HIV services for adolescents in sub-Saharan Africa.

## Methods

This article is based on desk research. It outlines international and regional human rights principles and norms applicable to adolescents in the context of health and HIV in Africa (Table 1). The human rights framework provides the basis to identify key public health principles relating to access to HIV testing, counselling, disclosure and treatment for adolescents as provided under the WHO guidance on adolescents (Box 1). These human rights norms, principles and public health frameworks are employed to review the normative content of the 28 HIV-specific laws adopted in sub-Saharan Africa as of 31 March 2017 (Table 2). This review focuses on HIV-specific laws because in countries where they exist, HIV-specific laws are much more likely to address access to HIV and health services for children and adolescents as opposed to other existing laws that are often silent on these issues [10].

**Box 1.** Key public health recommendations relating to independent access to HIV services for adolescents in the WHO Guidance

**Age of consent to HIV testing** Countries should consider best approaches within their legal and social contexts to lower the age of consent to HIV testing and counselling (p 20). Adolescents who have not reached the set age of consent but have reached sufficient level of maturity and understanding should be allowed to consent to HIV testing (p x). **HIV counselling** In the context of HIV testing and counselling, pre- and post-test counselling are critical for adolescents with or without HIV (pp 15 and 19). **Confidentiality and disclosure of HIV results** Adolescent services must be confidential. Disclosure should be done with the consent of the adolescent tested (p 47). Decisions concerning to whom to disclose test results should be made with the support of the provider or counsellor and a family member or friend if possible (p 47). **Consent to HIV Treatment** Adolescents who legally are given the right to access HIV testing, and counselling services should also have autonomous access to HIV prevention and treatment services (p 12). World Health Organization. HIV and adolescents: guidance for HIV testing and counselling and care for adolescents living with HIV: recommendations for a public health approach and considerations for policy-makers and managers. Geneva: Word Health Organization; 2013.

HIV-specific laws in sub-Saharan Africa were collected from January 2014 to March 2017 by searching peer-reviewed publications, reports and other materials relating to HIV and the law for any indication of the existence of such laws in these countries. This involved a search of existing databases of HIV-related laws and policies, such as ILO/AIDS, UNESCO HIV and health education clearinghouse and AIDSPortal, as well as compendiums of HIV-related legal materials and publications relating to HIV laws and policies in Africa [11–15]. In addition, a systematic Internet search using different combinations of the names of all sub-Saharan African countries and the words “HIV laws” and “HIV legislation” was used to retrieve information relating to the existence of HIV-specific legislation in specific countries. Where possible, official versions of the laws were secured through government gazettes, websites of national parliaments and other national and regional online repositories of laws.

A thematic analysis of the content of the 28 HIV-specific laws ascertained whether they address HIV testing, counselling and treatment for adolescents. HIV-specific laws without explicit provisions on adolescents were thus identified and marked as such. Those laws that contained provisions on adolescents were systematically reviewed through a data extraction and coding tool that described how they address key issues relating to age of consent to HIV testing, counselling, confidentiality and disclosure and HIV treatment for adolescents [16]. This tool consisted of an excel table with categories relating to the legal and policy areas of importance and concern for children and adolescents (excerpts from the tool are presented in tables below). The legal analysis was used to identify those HIV-specific laws that set an explicit age of consent. This age provided under these laws was noted in ascending order (i.e. from the lowest to the highest). In countries where no age of consent was explicitly set, the analysis involved reviewing and searching the HIV laws for expressions such as “child”, “children” and “minors” which may be used as alternative to specific ages. Where such terms were identified, we searched for and analysed the definition in the HIV law or explicit reference to another legislation defining the terms. We also reviewed HIV-specific laws for the use of the term “maturity” or similar notions as conditions for allowing access to HIV services for adolescents below the set age of consent, as recommended by the WHO Guidance. We further reviewed the laws for any specific provision relating to HIV counselling or protection of confidentiality for adolescents. Finally, we searched HIV-specific laws for provisions on access to independent HIV treatment for adolescents.

In addition to the general analysis of the 28 HIV-specific laws, the study also reviewed existing policy documents on access to HIV testing and counselling for adolescents in Burkina Faso, Chad, Kenya and Tanzania. This analysis compares the provisions of HIV-specific laws in these countries with national policies on HIV testing and counselling. The four countries were selected because the development of their HIV policy took place after the passing into law of the HIV-specific legislation and as a result should comply with the legislative stipulation. Furthermore, these countries represent all four sub-regions of sub-Saharan Africa and can illustrate the situation in the region more broadly.

## Results

Three of the 28 laws reviewed (Burundi, Chad and Equatorial Guinea) are silent on all aspects of HIV testing, counselling and treatment for children, adolescents or minors [17–19]. In addition, the HIV law of Cape Verde only provides that HIV test results of minor can be provided to their parents without any further details on whether minors can independently access HIV testing, counselling or treatment. The remaining 24 HIV-specific laws address one or more of the four public health recommendations provided in the WHO Guidance in relation to access to independent HIV testing and counselling and care for adolescents. However, none of these laws addresses all four recommendations. Some 11 countries explicitly set an age of consent for access to HIV testing services ranging from 11 years to 18 years (Table 3). Of these, only seven allow for independent consent to HIV testing below 18 years. Some 13 countries do not provide for an explicit age of consent to HIV testing but rather exclude “minors” or “children” from independent access to HIV testing. This means that in these 13 countries, only those who have reached majority or adults can consent to HIV testing (Table 3). The notions of “minors” or “children” are often not defined in the 13 HIV laws, thus leaving it to other provisions to determine the age of majority for independent access to HIV services (Table 3). These laws are also unclear about the type of majority foreseen for the purposes of HIV testing, notably whether it is legal majority or majority for sexual acts which are different in the laws of many countries [44].

**Table 3.** Age of consent to HIV testing in HIV-specific laws in sub-Saharan Africa

Age of consent to HIV testing	Countries
(a) Explicit age of consent	11 countries: Burkina Faso [20], Congo [21], Côte d’Ivoire [22], DRC [23], Gambia [24], Guinea [25], Kenya [26], Mozambique [27], Niger [28], Senegal [29], Uganda [30]
11 years	Mozambique (Article 23(3)) [27]
12 years	Uganda (Sections 1 and 10) [30]
14 years	Guinea (Article 22) [25]
15 years	Congo (Article 18) [21], Senegal (Article 12) [29]
16 years	Côte d’Ivoire (Article 4) [22], Gambia (article 14(1)(b)) [24]
18 years	Burkina Faso (Article 2 & 9) [20], Kenya (Sections 2 & 14) [26], DRC (Articles 2(4) & 37) [23], Niger (Articles 2 & 5) [28]
(b) Reference to “minors” or “children”	13 countries: Angola [31], Benin [32], Central African Republic [33], Comoros [34], Guinea Bissau [35], Liberia [36], Madagascar [37], Mali [38], Mauritania [39], Mauritius [40], Sierra Leone [41], Tanzania [42], Togo [43]

In eight countries that have set the age of consent in their HIV laws as 18 years or above, adolescents can still independently consent to HIV testing if they are considered to have reached sufficient maturity or fall into certain circumstances (Table 4). In three of these countries (Comoros, Mauritius and Togo), the law refers explicitly to the notion of “sufficient maturity” to allow access to HIV testing for adolescents below 18 years (Table 4). In these three countries, the law does not define the notion of sufficient maturity.

**Table 4.** Maturity and other circumstances enabling access to HIV testing for adolescents below the age of consent in HIV-specific laws

Maturity and other circumstances	Eight countries (Comoros, DRC, Kenya, Madagascar, Mauritius, Niger, Sierra Leone and Togo)
Sufficient maturity	Comoros (Article 18) [34], Mauritius (Section 7(5)) [40], Togo (Article 6) [43]
Emancipated minor	Comoros (Article 18) [34], Madagascar (Article 5) [37]
Pregnant	Kenya (Section 14) [26], Sierra Leone (Section 29(1)(b)) [41]
Married	Kenya (Section 14) [26], Madagascar (Article 5) [37], Niger (Articles 2 & 5) [28]
Parent	Kenya (Section 14) [26], Sierra Leone (Section 29(1)(b)) [41]
At risk of HIV infection	Kenya (Section 14) [26], Sierra Leone (Section 29(1)(b)) [41]
Best interest of the child requiresindependent testing	Comoros (article 18) [34], DRC (Article 37) [23], Madagascar (Article 5) [37]

HIV-specific laws also refer to various other notions and circumstances - intended to reflect sufficient maturity - to grant access to HIV testing for adolescents. They include whether the adolescent is an emancipated minor (Comoros and Madagascar), pregnant (Kenya and Sierra Leone), married (Kenya, Madagascar and Niger), a parent (Kenya and Sierra Leone) or at risk of HIV infection (Kenya and Sierra Leone) (Table 4). Some countries allow for several of these circumstances to apply while others only allow for one. Three countries (Comoros, DRC and Madagascar) refer to the best interests of the child for granting independent access to HIV services for adolescents below the set age of consent. However, the notion of best interests of the child is not defined in these laws.

Only two countries (Guinea Bissau and Mali) have specific provisions in their HIV legislation on HIV counselling for adolescents [35,38]. Yet in Guinea Bissau and Mali, these provisions are of very limited value because the HIV laws do not allow for independent HIV testing for adolescents in the first place. Madagascar is the only country that addresses access to independent HIV treatment for adolescents. In terms of Article 13 of the HIV law of Madagascar, children may access HIV treatment and care without parental consent where it is in their best interests or in the case of emancipated minors.

Only five out of all countries with HIV-specific laws that allow for HIV testing for adolescents below 18 years (either through lower age of consent or maturity) have provisions explicitly ensuring confidentiality and protection against disclosure of HIV test results. These are Congo, Gambia, Kenya, Sierra Leone and Uganda. This means that in the other countries, the HIV law may ensure independent access to HIV testing below 18 years, yet it does not protect against unauthorized disclosure of HIV test results of adolescents to parents, guardians or other caregivers.

In the four countries (Burkina Faso, Chad, Kenya and Tanzania) where HIV testing policies were reviewed against the provisions of HIV-specific laws, there are discrepancies between the age of consent for independent access to HIV testing (Table 5). In Burkina Faso, the HIV policy introduces exceptions that permit independent access to HIV testing contrary to the provisions of the HIV Act. In Kenya, the age of consent provided in the HIV policy is lower than that provided in the HIV law at 15 years compared to 18 years in the HIV law. In addition, the HIV policy of Kenya allows all children below 15 to independently consent to testing under exceptional circumstances. In Tanzania, the HIV testing policy provides for exceptional cases of access to HIV testing and counselling services for adolescents which are not recognized under the HIV law. In the case of Chad, both the HIV law and the HIV testing policy are silent on access to HIV testing for adolescents, and the HIV policy merely refers to majority for independent access.

**Table 5.** Comparing HIV testing provisions in HIV-specific laws and national HIV testing policies in four sub-Saharan African countries

		Country			
Issue	Normative source	Burkina Faso	Chad	Kenya	Tanzania
Age of consent	Law	18 years	Not available	18 years	Refers to child (age not defined in HIV law)
	Policy	18 years	Refers to minor (not defined) [45]	15 years [46]	18 years [47]
Maturity and other circumstances for independent access for adolescents below the set age of consent	Law	Not available	Not available	Below 18: – Pregnant – Married – Parent – At risk of HIV infection	Not available
	Policy	15–18 years if: – maturity – Married – Pregnant [48]	Not available	Below 15 years if: – married, – a mother/father of a child – otherwise no longer dependent on the parents [46]	Below 18 if: – married – have children – sexually – active [47]

## Discussion

This study shows that provisions in HIV-specific laws relating to HIV testing, counselling and treatment for adolescents are generally inadequate as they fail to take into account human rights principles and public health recommendations from the WHO Guidance. None of the countries with HIV-specific laws has adopted all four recommendations aimed at ensuring appropriate access to HIV services for adolescents. Overall, countries with HIV-specific laws can be divided into those with no provisions relating to independent access to HIV services for adolescents, those that explicitly exclude independent access to HIV services for adolescents and those that contain progressive provisions enabling some form of independent access to HIV testing, counselling and treatment for adolescents.

The fact that only 7 out of 28 countries reviewed have lowered the age of consent in their HIV-specific laws below 18 years is concerning because reducing the age of consent is one of the key guarantees for independent access to HIV services. Lowering the age of consent removes the discretion of healthcare workers which in practice may lead to denial of services due to social constructs [4]. Age of consent adopted in these HIV-specific laws range from 11 to 18 years raising questions on the reasons and criteria for adopting a specific age of consent in each country. In general, the age of consent seems to reflect special circumstances and agreements reached in each context based on the actors involved in the law-making process and their ability to effectively advocate in favour of adolescents’ rights and health. From this perspective, the lack of clear direction to countries in the WHO Guidance for setting the age of consent represents a weakness as it is likely to perpetuate inconsistency across countries in the norms relating to access to HIV services for adolescents [4].

The recognition in several countries of sufficient maturity as a criterion for an independent access to HIV testing for adolescents as provided in the WHO Guidance note is welcomed. However, these provisions often raise questions. First, the notion of “maturity” is not defined under the laws. In some countries, several circumstances have been provided under the law which may imply a reference to maturity. These include reference to adolescents who are pregnant, married, parents or at risk of HIV infection. However, such notions are narrow and may in practice lead to denying HIV services to many adolescents who are mature enough “to understand the meaning and consequences of HIV testing” but may not be married or pregnant. The introduction of the notion of the “best interests of the child” in Comoros, the DRC and Madagascar for access to HIV testing services for adolescents is a positive development. However, this notion of best interest needs to be clearly defined through regulations in a manner that effectively allows access to HIV testing for children and adolescents.

Generally, the adoption of lower age of consent and maturity and other exceptions in HIV-specific laws mostly apply to adolescents’ access to HIV testing. Almost no attention has been given in these laws to access to age-appropriate and adolescent-sensitive HIV counselling services. Similarly, all the laws are silent on access to independent HIV treatment for adolescents except in Madagascar. Even countries that have lowered the age of consent to allow autonomous HIV testing for adolescents do not provide for an independent access to HIV treatment. This situation contradicts the WHO Guidance which provides not only for adolescents’ independent access to HIV testing but also to counselling, treatment and care services.

These serious discrepancies in HIV laws are likely to undermine adolescents’ independent access to the full continuum of HIV services. This situation also can be expected to lead to confusion among healthcare workers who are mandated to provide independent access to HIV testing under HIV-specific laws yet cannot provide independent access to HIV counselling and treatment services for the same adolescents. As noted in the WHO Guidance, HIV testing is not “an end in itself”, but an entry door to comprehensive post-test services for all adolescents [4]. Similarly, the fact that HIV-specific laws do not address access to other sexual and reproductive health services for adolescents - including prevention services adapted to their needs - is a missed opportunity for the HIV response and for public health.

The study further highlights contradictions between HIV-specific laws and policy documents relating to HIV testing for adolescents. These include discrepancies between age of consent in HIV laws and in policy documents, as well as differences in the circumstances for independent access below the set age of consent. This situation is likely to lead to confusion among healthcare providers and adolescents seeking HIV services. While healthcare workers at facility level are more likely to be aware of, and to apply, the provisions of policies relating to HIV testing, it is expected that conflict of norms between HIV laws and policies on the age of consent to HIV services will negatively impact their willingness or ability to provide services to adolescents.

Although the provisions of HIV-specific laws apply in principle to all adolescents, experiences and evidence from across Africa show that adolescents who belong to key populations, particularly those whose sexual practices, gender identity, life choices and circumstances are criminalized, may not enjoy the protections provided by these laws [4–6]. The fact that HIV-specific laws are often silent on key populations may further compromise the application of enabling HIV testing and counselling provisions in HIV-specific laws to young key populations [49].

## Key recommendations

The 2016 Political Declaration on HIV and AIDS endorsed a Fast-Track approach to the HIV response aiming to accelerate and expand access to HIV prevention, testing and treatment for all, including for adolescents and young people [50]. Efforts by HIV stakeholders to expand access to HIV services for adolescents in sub-Saharan Africa should thus address national laws and policies that represent barriers for adolescents. In all countries, this will require creating enabling political, social and cultural environments that supports access to HIV and health services for adolescents including through sensitization and engagement of legislators, health authorities, opinion leaders, communities and young people. In countries where HIV-specific laws have been adopted, specific approaches for addressing barriers and for facilitating access to HIV services for adolescents should be based on the content of these laws and they should involve the following:

### Where HIV-specific laws are silent on adolescents’ access to HIV services

Countries should adopt appropriate measures to facilitate access to services through law reform or through regulations that do not require parliamentary processes. These reform efforts should be based on best available public health evidence and human rights standards as provided under the WHO Guidance. The Children’s Act of South Africa is a best practice that could be considered by countries (Box 2).

**Box 2.** Provisions on independent HIV testing, counselling and treatment for children in South Africa

**Independent consent to HIV testing (Section 130(2))** “Consent for a HIV-test on a child may be given by- (a) the child, if the child is- (i) 12 years of age or older; or (ii) under the age of 12 years and is of sufficient maturity to understand the benefits, risks and social implications of such a test” **Counselling before and after HIV-testing for children (Section 132)** “(1) A child may be tested for HIV only after proper counselling, by an appropriately trained person, of- (a) the child, if the child is of sufficient maturity to understand the benefits, risks and social implications of such a test; and (b) the child’s parent or care-giver, if the parent or care-giver has knowledge of the test. (2) Post-test counselling must be provided by an appropriately trained person to - (a) the child, if the child is of sufficient maturity to understand the implications of the result; and (b) the child’s parent or care-giver, if the parent or care-giver has knowledge of the test”. **Independent consent to treatment (Section 129(2))** “A child may consent to his or her own medical treatment or to the medical treatment of his or her child if- (a) the child is over the age of 12 years; and (b) the child is of sufficient maturity and has the mental capacity to understand the benefits, risks, social and other implications of the treatment”. *Abstracted from Children’s Act 2005 of South Africa, No 38 of 2005.*

### Where HIV-specific laws explicitly exclude or limit independent access for adolescents

These are legal barriers that should be removed. Reform should focus on lowering the age of consent for independent access to HIV testing, counselling and treatment as well as for other prevention and sexual and reproductive health services. Pending amendment and reform of restrictive provisions in HIV laws, regulations and policies should be adopted to enable independent access to HIV testing and treatment for children.

### Where HIV-specific laws have progressive and enabling provisions on access to HIV services for adolescents

Countries should ensure effective implementation of these enabling provisions, including through the adoption of guidelines where necessary. Education and sensitization on the enabling provisions should be prioritized together with training for healthcare providers on non-discriminatory, appropriate and ethical HIV testing, counselling and treatment of adolescents. Sensitization should also target the general public, youth-led organizations as well as parents and other caregivers on the content of the law and the importance of facilitating access to HIV services for adolescents.

## Conclusions

This study demonstrates that laws and policies relating to access to HIV testing, counselling and treatment for adolescents in sub-Saharan Africa remain gravely inadequate. While recognizing that various laws influence access to health and HIV services for adolescents, this study stresses that efforts to facilitate access to HIV services for this population in sub-Saharan Africa should address HIV-specific laws where they already exist. Restrictive provisions in HIV-specific laws should be reformed. Protective norms contained in the laws, such as lower age of consent and the recognition of maturity for access to HIV services, should be effectively implemented including by translating them into national HIV testing and treatment policies and ensuring sensitization and training of healthcare workers, communities, youth-led organizations and caregivers on the rationale and content of laws and regulations that enable access to HIV services for adolescents. Ultimately, this study reiterates the need for action in all countries across Africa and beyond to review their laws and policies to create an enabling environment to accelerate access to HIV prevention, testing and treatment services for adolescents.

## Abbreviations

ACHPR, African Charter on Human and Peoples’ Rights; ACRWC, African Charter on the Rights and Welfare of the Child; CEDAW, Convention on the Elimination of All forms of Discrimination Against Women; ICESCR, International Covenant on Economic Social and Cultural Rights; CRC, Convention on the Rights of the Child; GC, General comment; ICCPR, International Covenant on Civil and Political Rights; Maputo Protocol, Protocol to the African Charter on Human and Peoples’ Rights on the Rights of Women in Africa; UDHR, Universal Declaration on Human Rights.
